# Unfolding the dynamical structure of Lisbon’s public space: space syntax and micromobility data

**DOI:** 10.1007/s41109-021-00387-2

**Published:** 2021-06-30

**Authors:** Helena Freire de Almeida, Rui J. Lopes, João M. Carrilho, Sara Eloy

**Affiliations:** 1 Instituto Universitário de Lisboa (ISCTE-IUL), ISTAR, Lisbon, Portugal; 2 Iscte - Instituto Universitário de Lisboa, Lisbon, Portugal; 3 Instituto de Telecomunicações, Lisbon, Portugal; 4 CICANT, Universidade Lusófona de Humanidades e Tecnologias, Lisbon, Portugal

**Keywords:** Space syntax, Micromobility, Urban network analysis, Community detection, Variation of information

## Abstract

Space Syntax and the theory of natural movement demonstrated that spatial morphology is a primary factor influencing movement. This paper investigates to what extent spatial morphology at different scales (node, community and global network) influences the use of public space by micromobility. An axial map and corresponding network for Lisbon’s walkable and open public space, and data from e-scooters parking locations, is used as case study. Relevant metrics and their correlations (intelligibility, accessibility, permeability and local dimension) for the quantitative characterization of spatial morphology properties are described and computed for Lisbon’s axial map. Communities are identified based on the network topological structure in order to investigate how these properties are affected at different scales in the case study. The resulting axial line clustering is compared via the variation of information metric with the clustering obtained from e-scooters’ proximity. The results obtained enable to conclude that the space syntax properties are scale dependent in Lisbon’s pedestrian network. On the other hand both the correlation between these properties, the number of scooters and the variation of information between clusters indicate that the spatial morphology is not the only factor influencing micromobility. Through the comparative analysis between the main properties of the public space network of Lisbon and data collected from e-scooters locations in a timeframe, centrality becomes a dynamic concept, relying not only on the static topological properties of the urban network, but also on other quantitative and qualitative factors, since the flows’ operating on the network will operate several transformations on the spatial network properties through time, uncovering spatiotemporal dynamics.

## Introduction

The study of the city is a transdisciplinary endeavor. Since the complexity of cities seems to challenge any description, several simplified concepts of the city were used as describing models, suggesting that the dynamics of socio-spatial relations could follow clear hierarchies or regular geometries, or that parts could be separated from the whole emergent urban phenomenon (Hillier 2009). One of the major issues is to seek for other than linguistic concepts, more suitable to describe the dynamics of such a complex field, a common language for a deeper understanding of its dynamics.

To approach a research topic like human movement in the City, first it is necessary to understand what kind of problem we face. Jacobs (1993), following Warren Weaver’s paper entitled “Science and Complexity”, asserts that cities can only be understood as organized complexity problems. In fact, cities are often defined as systems of systems, built like living organisms. Its scope goes beyond buildings, roads and people. There is also a spacetime dimension where relations and activities happen, conditioning our perceptions and transforming the city (Batty 2013). As these infrastructural and social components are strongly interrelated (Bettencourt and West 2010; Barthelemy 2019), in this paper we propose a discussion about the city from the point of view of complexity studies, by analyzing the interrelations between the urban fabric (hard components) and the social dynamics (soft components) that characterize and transform urban life.

The research question under analysis is: Are the static components of the urban fabric the only factors conditioning movement patterns of micromobility vehicles in the urban system? This paper is part of an ongoing research project focused on this question, raising the hypothesis that beyond the built environment, spatiotemporal factors affect movement patterns in the urban fabric.

If spatial morphology is a primary factor influencing movement (Volchenkov and Blanchard 2007), as demonstrated by the theory of natural movement (Hillier et al. 1993), and cities are ‘mechanisms for generating contact’, their primary elements are the structure of the urban grid, distribution of land uses and building densities. The urban experience is therefore, according to the authors, the result of the combination of these three components, generating the ‘movement economy’ (Hillier 1996). Complementary to this triangular relation, other studies suggest that the articulation between existent urban fabric elements such as transport accessibility, street network and land use are also strongly related to pedestrian activity (Koohsari et al. 2019; Dhanani et al. 2010).

This study proposes an extension of this relation to spatiotemporal factors like events or activities. The goal of the paper is to present a comparative analysis of the main properties of the public space network of Lisbon: (a) as a spatial infrastructure and (b) as a structure for space-time flows.

Firstly, we present a theoretical framework on complexity and network science in the context of urban dynamics and discuss the importance of the analysis of quantitative and qualitative aspects of urban rhythms. This section serves as the foundation to propose spatiotemporal events as structuring elements of the ‘mechanism’ of cities, alongside with spatial morphology.

The case study is the walkable and open public spaces network of Lisbon. The network generation followed space syntax methodology, due to its capacity to study the relations between spatial organization and social behavior. It is composed by axial lines (vertices), which represent the longest lines of sight of each convex space, and intersections of these axial lines (edges).

In the next section we introduce the case study, the collected data, and the methods used for the development of the study. Space syntax axial map representation model will be used, which is defined by the topological relations between spaces, rather than Euclidean distance. A discussion about space syntax and graph theory measures is conducted at different levels. Collected data is analyzed independently and in confrontation with the spatial network by finding correlations with the space syntax measures.

Main results of the analysis suggest that, while the network as a communication channel reveals its primary properties, which influences movement patterns in the urban grid, the flows operating on the network transform these properties through time, uncovering other spatiotemporal conditioning factors.

## Background

The urban experience is made of complex correlations between activities, behaviors and perceptions, immersed in and conditioned by the urban fabric. The analysis and quantification of these correlations is extremely challenging. Both network science and the emergence of new sources of data on social and human activities allow to mitigate these difficulties as they provide both tools and data for studying the organizing principles of these interaction networks (Barabási and Pósfai 2016). These are composed of information, communication and activities’ flows, even if they are characterized by an irregular and dynamic structure. In fact, Batty (2013) argues that “to understand place, we must understand flows, and to understand flows we must understand networks”, as the ultimate goal of networks is to define relations between the objects that comprise our system of interest.

Network science research has revealed that the architecture of networks in various domains of science and nature are governed by the same organizing principles, which allows the use of a common language and mathematical tools to explore them. In fact, graph theory and network science have been extensively used, along with Geographic Information Systems (GIS), and data from transport, shared mobility and social network services to analyze urban morphology, its dynamics and develop urban mobility models (Dhanani et al. 2010; Guo et al. 2017; Yildirimoglu and Kim 2017; Boeing 2019; Gallotti et al. 1993).

Micromobility flows and public space are just two examples of the vast number of layers in an urban system. Infrastructural and social components are so strongly interrelated, one can make multiple analyzes at different levels, in order to reveal their unique properties and relations. In this paper, we focus on the interrelations between the built environment and the social dynamics that characterize and transform urban life.

The notion of rhythm is deeply embedded in human perception. LeFebvre, in his ‘Rhythmanalysis’ essay (LeFebvre 2004), distinguishes quantitative aspects and elements, which mark time and distinguish moments, and qualitative aspects, which ground and link them together. It is through the perception of cycles and patterns that we measure time and space. Therefore, urban dynamics is closely related to the analysis of quantitative and qualitative elements of rhythm, as each of its components has its own dynamics, revealed by spatiotemporal patterns and cycles, a continuous, but not synchronous, flow of information and activities.

Batty (2018) distinguished two types of urban rhythm: high frequency, operating at the level of human time frames (seconds, days, months), and low frequency rhythms, operating over longer periods, sometimes several years or generations.

Nowadays, some former low frequency urban pulses are turning into high frequency, or at least increasingly working at both timeframes. Pedestrian movement is one of them. With the spreading of smart phones’ apps, associated to shared soft mobility devices, mode commuting is increasingly easier, from walking to shared bicycles or electric scooters, along with subway or bus. Each mode contributes to support mobility in the city, producing a multiplicity of rhythms on the traditional street network and transforming it.

To approach the research question, this study applies space syntax theory to investigate the relations between human behavior and the urban form, as well as complex systems tools, namely network science tools, community detection algorithms and information theory metrics (notably variation of information).

## Data and methods

Two sources of data were used, namely cartographic data, for the axial map generation, and micromobility dynamic data on the network, collected from the number and location of e-scooters during one week. This data was used as an activity indicator for each axial line of the network.

In this study we describe and relate variations of e-scooters locations through time to the network measures and community structure of the static and physical components of the urban system.

A comprehensive analysis of the network requires a multiscale approach, namely at the network (macro), community (meso) and the node (micro) levels. This approach helped us on detecting structural properties and morphological interscalar coherence of the spatial network. At the macro level, a study of the global structure of the network is conducted, regarding its size, order, density, degree distribution and assortativity.

At the meso level, we detected network communities and identified the geographical correspondence, looking for association of network topological communities with identifiable local areas of urban livability.

At the micro level, we analyzed the nodes of the network in terms of centrality measures.

Data collection of e-scooters locations through time was also subject to cluster partition analysis, combining the k-means clustering algorithm with the elbow strategy to determine the optimal number of clusters and k-means++ to find the initial values (seeds) (Arthur and Vassilvitskii 2007; Syakur et al. 2018). Next, we compared the network community structure with the k-means partition for each moment of data collection, using the variation of information metric (Meila 2007). The e-scooters locations were then assigned to a network community, by finding the nearest axial line to each GPS location. Space syntax measures were then compared to e-scooters average concentration in each community. Finally, we applied a comparative analysis of the fluctuations of e-scooters locations in the axial lines with higher values of each space syntax measure.

### Study network and data

**Fig. 1:**
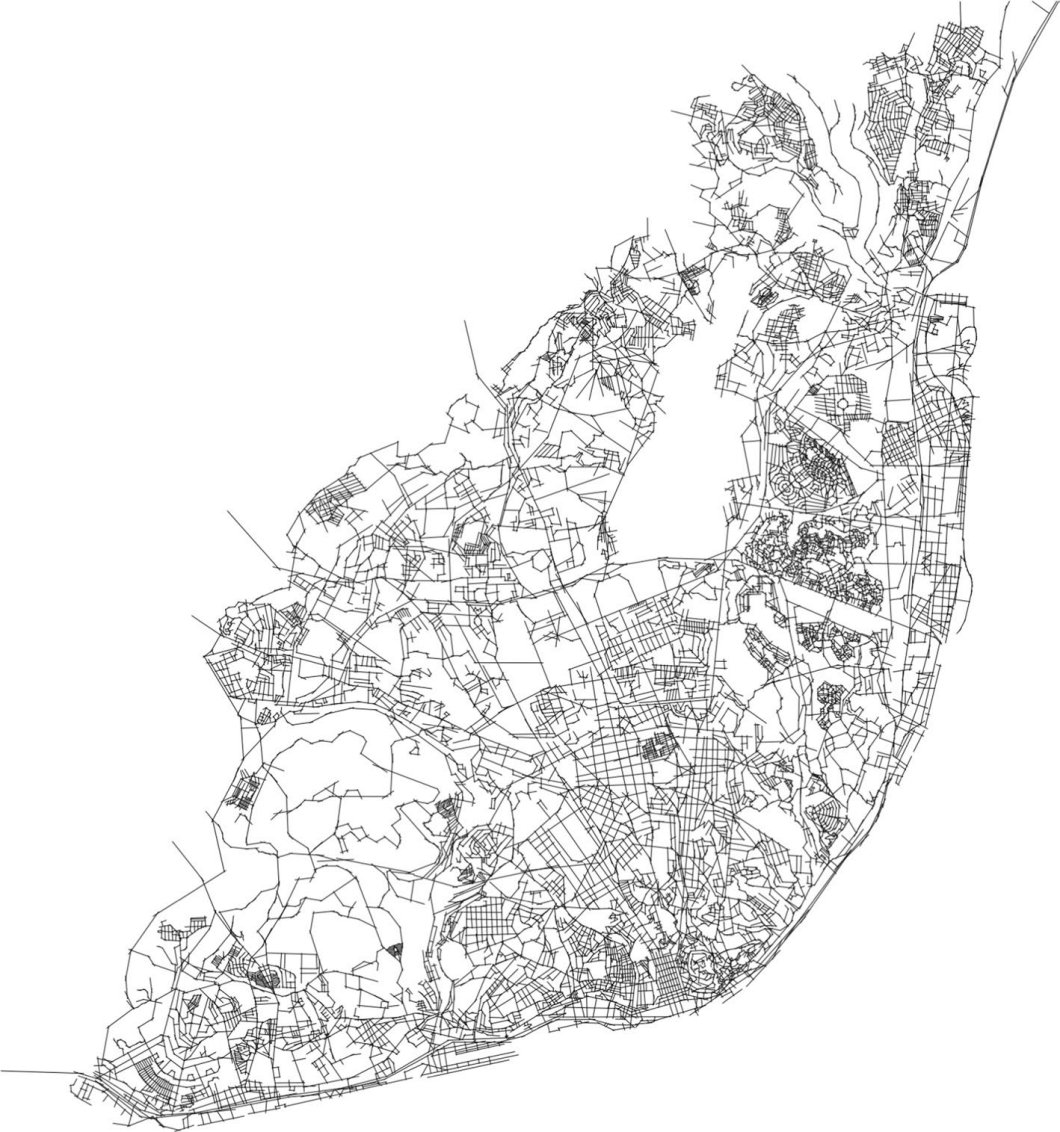
Axial map. Representation of Lisbon’s walkable open public spaces using space syntax methodology

The network under study is the walkable and open public spaces network of Lisbon. The network was generated from the available military cartographic data from the Instituto Geográfico do Exército, where open public spaces accessible for pedestrians were selected and drawn in a computer aided-design (CAD) software. The representation model followed the space syntax axial map methodology, by decomposing a space map into a complete set of intersecting axial lines, which are the fewest and longest set of straight lines of sight which passes through each convex space and makes all axial links (see Fig. 1). It is a process of representing the relation between spatial organization and social behavior, since it describes how one can understand and move around a certain spatial configuration.

In spite of being a fundamental tool in space syntax, the method for generating the axial maps has been criticized for being time consuming and subjective. To overcome this difficulty, other methods have been developed, as generating the axial lines through street center lines, using the Gestalt principle of good continuity (Turner 2007; Liu and Jiang 2012). The choice of using axial lines as the representation model is related to their ability to describe the whole open public space area where pedestrians can freely move.

The choice of what would be the most appropriate network to analyze micromobility patterns lied on the following reasons: (a) the observable fact that micromobility vehicles like e-scooters, similarly to pedestrians, use mainly these spaces in the urban fabric; (b) the low speeds (below 25 km/h) of e-scooters do not change significantly, when compared with pedestrians, the available time for decision-making of changes in direction along a route, nor the spatial perception.

**Fig. 2:**
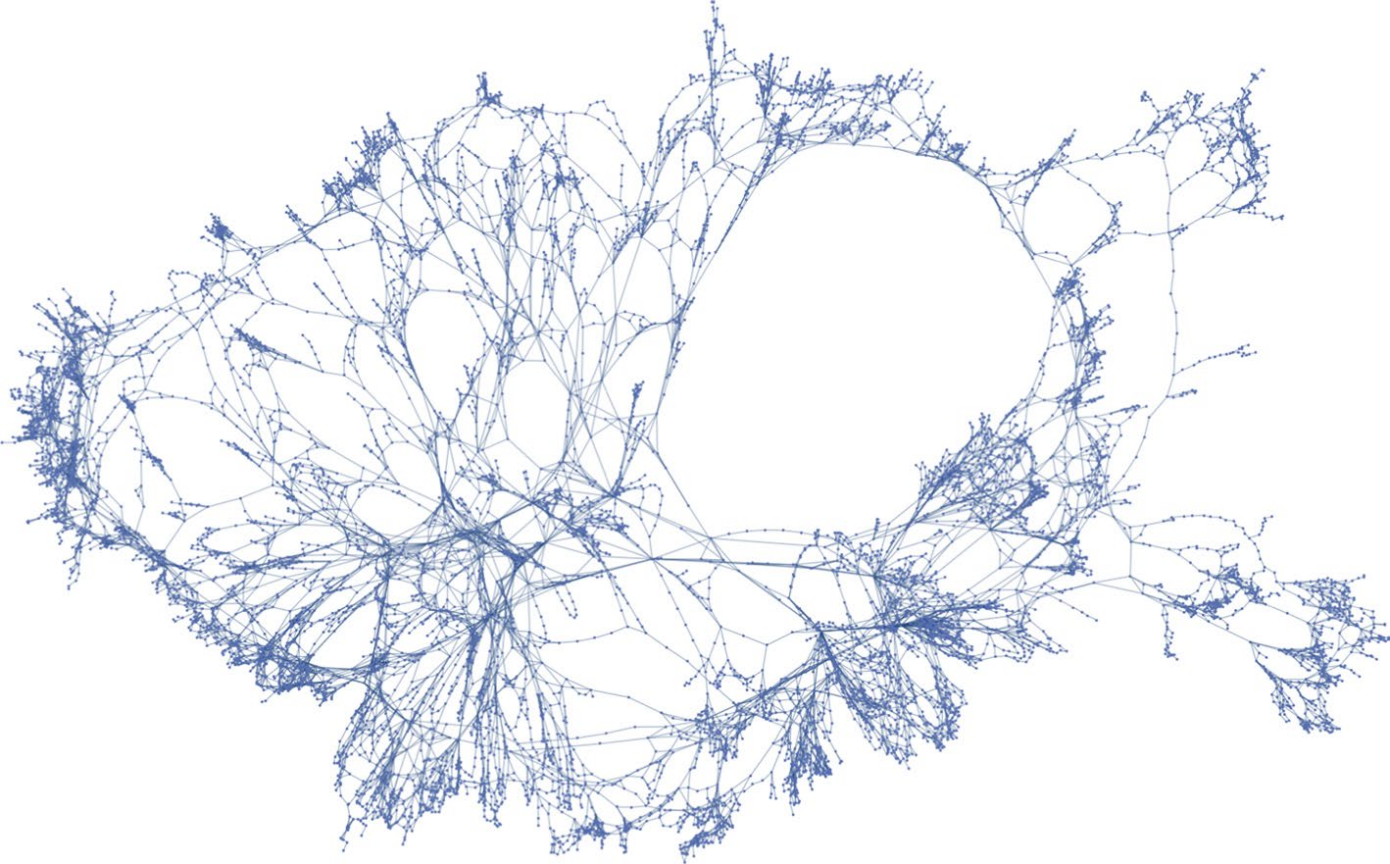
Graph representation. Resulting graph from the axial map of Lisbon

Axial lines represent spaces and are the vertices of the graph, while the intersections between axial lines represent the spatial junctions and correspond to the graph edges (see Fig. 2). This transformation leads to a non-planar dual graph, which describes the perceived hierarchy and structure of the urban grid (Volchenkov and Blanchard 2007).

The fundamental relation in axial maps is space adjacency. When two spaces are adjacent, it means one can freely move between them without obstacles. The studied network is undirected, as it describes the spatial relations from the point of view of pedestrians, or micromobility vehicles, and connected, as the nature of the network in study is to be fully accessible for pedestrian and micromobility vehicles.

The space syntax method calculates and describes topological inequalities in the public space network. Its contribution allowed for a deeper understanding about the relationship between space and society (Hillier et al. 1993; Hillier 1996, 2009; Volchenkov and Blanchard 2007). This method comprises a set of theories, including the well known theory of natural movement, which states that the degree of a public space network integration influence the movement flow (Penn et al. 1998; Koohsari et al. 2019; Yamu et al. 2021). The precision of the spatial elements used, high level of falsifiability and testability and high levels of empirical support of this method allowed its application on a large variety of built environments, and to strengthen and develop descriptive theories on urban space (Yamu et al. 2021).

### Network description and measures

The axial map lines coordinates were imported to Wolfram Mathematica and intersections were determined to define the graph. The software used for graph analysis was also Mathematica, enhanced with the IGraph toolkit. For the coordinates system transformation and axial map calibration we used Geopandas Pyhton package.

The axial map is represented by the graph with *V* vertices, corresponding to axial lines, and *E* edges, correspond to the intersections between axial lines. This graph is coded using the adjacency matrix $A_{G}$ with elements $a_{ij}$, where $a_{ij}=1$ corresponds to axial lines *i* and *i* being adjacent and $a_{ij}=0$ otherwise. At the macro level we describe the global structure and topology of the network, using the measures of size, order and density, as well as the global network degree distribution and assortativity. Both density, assortativity coefficient and degree distribution are good indicators of the topology of the network. Usually, public space networks are sparse, but present some level of assortativity, which relates to areas of intense local life, naturally perceived urban units, or neighborhoods.

These macro level measures are computed in the standard way:

- Order:
  1 $$\begin{aligned} N=\left| V\right| \end{aligned}$$
- Size:
  2 $$\begin{aligned} M=\left| E\right| \end{aligned}$$
- Density (undirected network):
  3 $$\begin{aligned} \dfrac{2 \dot{M}}{N\dot{(}N-1)} \end{aligned}$$

The assortativity coefficient, $AC_{G}$, indicates the likelihood of nodes with the same degree to be adjacent, and can be computed via the Pearson correlation coefficient of degree between pairs of linked nodes *i* and *j*. We compute this value using the process proposed in Newman (2002), where $q_k =\frac{(k+1)p_{k+1}}{\sum _j{jp_j}}$ is the remaining degree distribution and $p_k$ is the degree distribution as defined in Eq. 5. In Eq. 4$e_{jk}$ is the joint distribution of the remaining degree for a randomly chosen node pair in the graph, and $\sigma _q^2$ the variance associated to $q_k$.

4 $$\begin{aligned} AC_{G}=\frac{1}{\sigma _q^2}\sum _{ij}(ije_{ij}-q_iq_j) \end{aligned}$$

The degree distribution is the probability of a randomly selected node of the graph has degree *k* (Barabási and Pósfai 2016),

5 $$\begin{aligned} p_k=P_G(k)=Pr(d_i=k|i \in V) \end{aligned}$$

It is expected that the degree distribution may provide further evidence on the presence of hubs, that may have been indicated by the assortativity coefficient.

At the micro level, i.e., when considering each axial line, space syntax presents four measures that were conceived to describe its global and local properties in the network. The properties of each axial line in the global network are described using commonly accepted measures in space syntax theory. These syntactic measures are strongly related to those used in graph theory. However, space syntax makes some adjustments to adapt graph theory measures to the studied object, namely a network of urban convex spaces (that can be totally visible from an individual who is inside the network).

The first local property is connectivity; it is defined as the number of immediate neighbors that are directly connected to a space (Klarqvist 1993), corresponding to the graph theory node degree metric.

6 $$\begin{aligned} Connectivity(i)=d_i=\sum _{j=1}^{N}a_{ij} \end{aligned}$$

The second local measure is the axial line control value (CV). This measure quantifies the degree to which a space controls access to its immediate neighbors, *N*(*i*) considering the number of alternative connections that each of these neighbors has (Klarqvist 1993), and it is determined through the following formula (Volchenkov and Blanchard 2007):

7 $$\begin{aligned} CV(i)=\sum \limits _{j\in N(i)}{\frac{a_{ij}}{d_j}} \end{aligned}$$

Global measures are based on depth, which is the topological distance between node pairs in the graph *G*, or the least number of syntactic steps that are needed to reach one node, *j*, from another one, *i* (Klarqvist 1993). This corresponds to $d_ij$ the shortest path length between *i* and *j*. Shortest paths between node pairs are discovered using Dijkstra’s algorithm.

For each node, i.e., axial line, one computes its mean depth via Eq. 8:

8 $$\begin{aligned} Mean Depth(i)=l_i=\sum \limits _{j=1}^{N}{d_{ij}} \end{aligned}$$

The first global property is integration, a static property which describes the mean depth of a space to all others of the system (Klarqvist 1993). It is an indicator of the level of segregation of a space from the whole network (global integration) and can also be thought as the potential to-movement, or the tendency of a space to be a destination (Hillier 2009). This measure is determined by the inverse of Real Relative Asymmetry (RRA),

9 $$\begin{aligned} RRA(i)=2\frac{l_i-1}{D_N(N-2)} \end{aligned}$$

which compares the mean depth $(l_{i})$ for node *i* with the mean depth of the root nodes in a diamond shape graph (Kruger 1989; Kruger and Vieira 2012) according to the following normalization parameter:

10 $$\begin{aligned} D_N=2\frac{N(log_2(\frac{N+2}{3})-1)+1}{(N-1)(N-2)} \end{aligned}$$

Another global measure commonly used in space syntax is global choice, which measures the flow through a space, or a node of the graph *G* (Klarqvist 1993). It is strongly related to the graph theory measure betweenness centrality, as it captures the potential of each node to be used in information transfer (Barabási and Pósfai 2016), and can be interpreted by the movement flows in a spatial network. The global choice, normalized by the number of vertex pairs used in the shortest path calculation, can be determined by the following formula:

11 $$\begin{aligned} Choice(i)={\sum \limits _{s,t \in V, i \ne s, i \ne t}{\frac{\sigma _{sit}}{\sigma _{st}}}}, \end{aligned}$$

where $\sigma _{sit}$ is the number of shortest path between each pair *s*, *t* that transverse *i*. The normalization value $\sigma _{st}$ accounts for the total number of shortest path between *s* and *t*.

Movement patterns inside a spatial network also depend on its structural cohesion. Hillier (1996) and Penn (2001) conjecture that human cognitive capacity may partly manifest in the form of detecting a correlation between perceived consistent variations of a local property and global properties of a spatial system. Second order measures are correlations between fundamental aspects of urban structure that present values above $R=0.50$, allowing the spatial structure to be readable, or intelligible. This cognitive relation between individuals inside the network and its structure is an important plank of the social theory at the basis of space syntax (Penn 2001). Second order measures (intelligibility, permeability, accessibility and local dimension) are based on this argument and reveal cognitive properties of the urban structure.

Intelligibility can be measured by analyzing the relation between how a system can be perceived from its parts and what it is like in an overall pattern, that is, as a distribution of integration. It is expressed by a scattergram showing the degree of correlation between the connectivity of an axial line, which is a local property, and its integration, a global property which relates the line to the system as a whole (Hillier 1996).

Permeability is expressed by the correlation between the connectivity and the axial line length. This measure permits to understand to what degree the connectivity is more related to its length than to the actual porosity of a local area, while revealing the potential diversity of paths inside the urban fabric and along the line (Serdoura 2006).

Accessibility measures in what degree the spaces with higher control have good accessibility, and it is expressed by the correlation between global integration and control value (Serdoura 2006).

Local dimension measures the local sense of safety by correlating connectivity and control values. If lines with higher connectivity present also high control values, it generates the perception that the individual can visually control the surroundings from that space (Serdoura 2006).

In this paper, we compute and analyze these second order measures, at both network node and community levels.

### Community detection and meso scale analysis

The goal of this analysis is to divide the network into smaller areas with denser intra-connections, in order to capture morphological units within the system. This partition emerges from the public space structure itself, and does not necessarily coincide with administrative borders, historical or identity units of the system.

Although there have several different methods been proposed and developed, there is no definitive and unique approach on how to identify communities (Yildirimoglu and Kim 2017; Fortunato 2010; Newman and Girvan 2004). At the heart of this discussion, there is the basic concept of ‘modularity’, “a function which evaluates the goodness of partitions of a graph into clusters” (Fortunato 2010). The open public space networks are characterized by strong modular organization, reflecting functional associations between components (spaces). Global distribution of flows within the urban system and between systems is ensured by a sub-set of interconnected spaces that form the structural skeleton of the network. These spaces accommodate through-movement (Hillier 2009), linking highly intra-connected communities, which usually correspond to morphologically more coherent and homogeneous urban fabrics, the meso-scale elements of the spatial network, or neighborhoods. The modularity may vary for each network subject of analysis, due to several reasons, as the historical process of urban growth and planning, natural obstacles or administrative limits, but even in informal settlements we can find this characteristic.

**Fig. 3:**
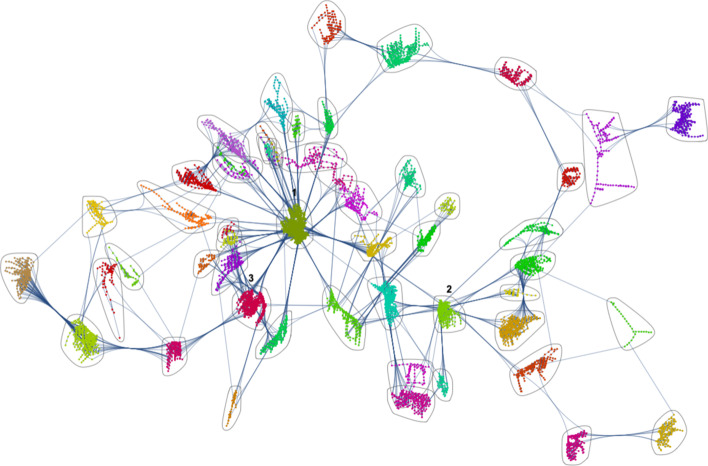
Community detection. Resulting community detection graph using fast greedy algorithm. Labeled communities: 1 - Av. Novas; 2 - Parque das Nações; 3 - Baixa-Santos

**Fig. 4:**
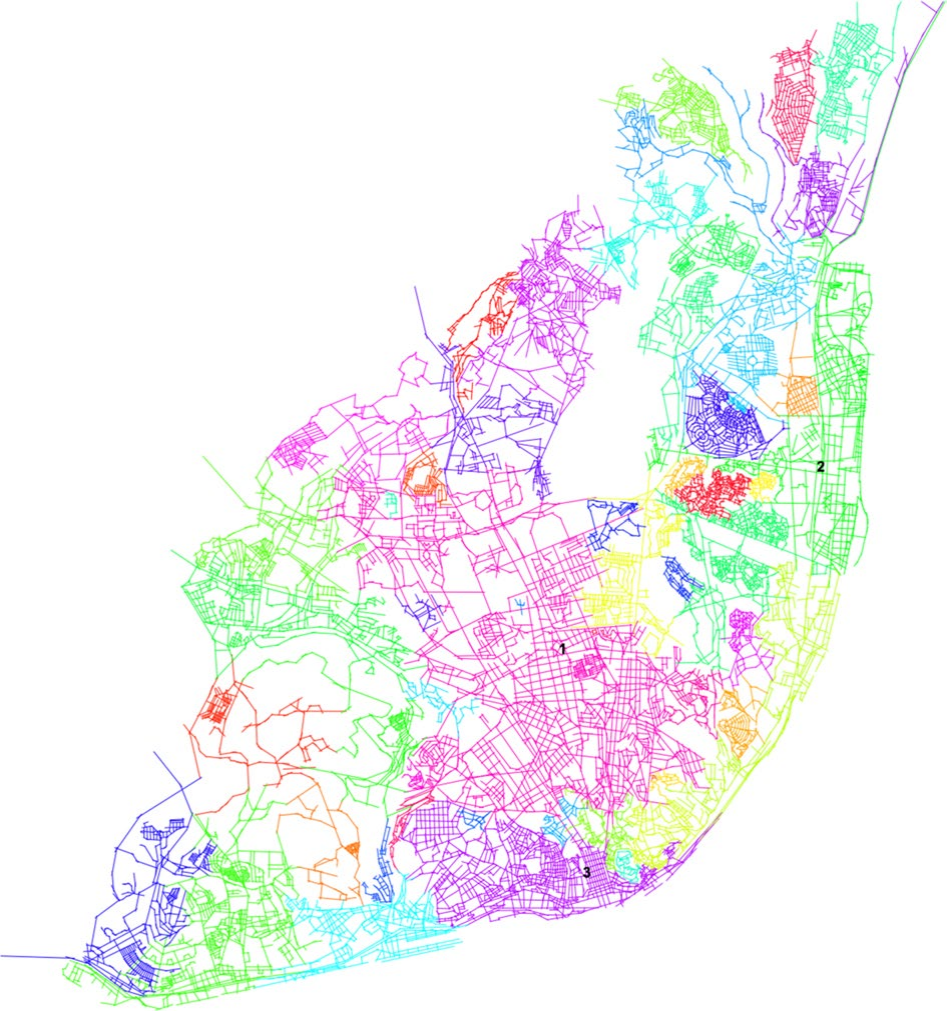
Geographical correspondence of communities. Communities representation on the axial map. Labeled communities: 1 - Av. Novas; 2 - Parque das Nações; 3 - Baixa-Santos

We selected the fast greedy algorithm (Newman 2004; Clauset et al. 2004; Orman and Labatut 2009) to perform community detection, due to its improvements on computational time and adequacy to large sparse networks that present hierarchical structure (see Fig. 3). The geographical correspondence of each community can be visualized in Fig. 4.

According to natural movement theory, spatial morphology is a primary factor influencing movement, therefore the detected communities should accommodate to-movement (Hillier 2009), neighborhood level, pedestrian activities and short length trips by the population, and through-movement spaces, which correspond to the main traffic routes of the urban system, with the role of linking communities and the whole system with the neighbors.

This motivates a chief contribution provided by this paper: apply the previously described space syntax metrics, where communities are used as vertices instead of axial lines and the network edges are defined by axial line intersections linking neighbor communities.

### Temporal analysis

The data collected for micromobility flows analysis was extracted from the Bird Application Programming Interface (API), which supplied the GPS location of parked scooters (https://mds.bird.co/gbfs/system_information.json). The timespan of collection was from 14th December 2020 (Monday) to 20th December 2020 (Sunday), starting at 7AM to 23PM (every two hours). The ID of each e-scooter is anonymized, therefore we were not able to directly track the movement patterns of these vehicles. The collected data was subject to a geo-reference calibration in order to fit the axial map.

In order to assess the influence of spatial morphology in movement patterns, we compared the topological properties of the network and community structure with the variations on the number of parked e-scooters at each location. First, we applied the k-means algorithm for each day/hour of data collection, which is computationally time efficient on grouping large amounts of data. However, its sensitiveness to the number of clusters was overcome using the elbow strategy (Syakur et al. 2018). The starting values for each cluster location were defined via the seeding technique k-means++, which improves both speed and accuracy of k-means algorithm (Arthur and Vassilvitskii 2007).

Secondly, we compared the network and the data partitions through the variation of information (VI) technique, a metric criterion for cluster comparison derived from information theoretic principles (Meila 2007). High values of VI suggest that e-scooters tend to gather in clusters than do not fully coincide with the network community structure. For instance, a group of e-scooters can be found in an area that covers more than one community, or a network community can contain several different clusters of e-scooters. Next, we compared the second order measures with the average number of e-scooters per community, in order to seek for correlations of the use of public space with some properties developed in space syntax to quantify characteristics related to perception and behavior on public space.

Finally, at the node level, we analyzed the variation of the number of e-scooters through time in some of the most important nodes of the graph, assuming the variation of the number of e-scooters through time as a proxy for movement patterns, regarding that the locations of parked scooters correspond to the origin or destination of travels.

## Results and discussion

Following the transformation process of the axial map into a graph, the data was analyzed and visualized, which allowed: (a) the topological description of Lisbon’s open public spaces network; (b) its characterization through space syntax measures; (c) identification of the most structural important spaces, using different criteria; (d) to detect communities based on the structure of the network; (e) the comparison of space syntax measures before and after reducing the original graph into a communities graph; and (f) after e-scooters location retrieval through a timespan, it was possible to compare topological measures with the dynamical use of public space.

When we measure the observed network, we can see it is sparse, which is typically the case of real-world networks. Also, the diameter of the network tells us that the two most far vertices of the network are sixty two (62) lines of sight away from each other. The level of assortativity reveals a low positive coefficient, which means that there may be communities in the network (Table 1).

**Table 1:** Global network properties. Size, order, density and assortativity measures of Lisbon’s open public spaces network

Network measure	Value
Number of vertices (N)	10,067
Number of edges (M)	18,774
Diameter	62
Density	0.0004
Assortativity (AC)	0.1295

**Fig. 5:**
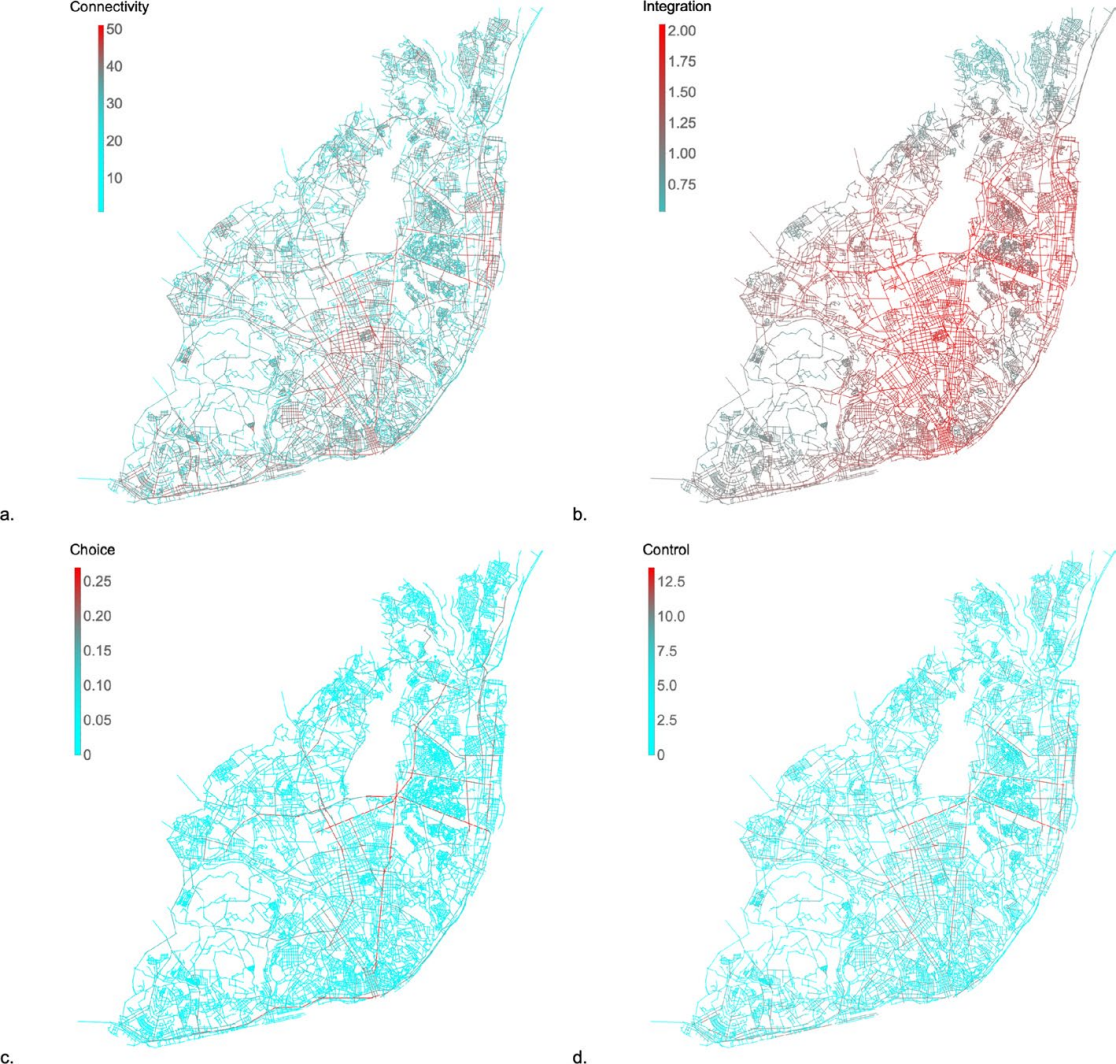
Space Syntax measures representation for the whole network. **a** Connectivity (node degree); **b** Global Integration (based on closeness centrality); **c** Global Choice (betweenness centrality); **d** Control value

We calculated each of the four space syntax measures presented for all the nodes of the network. The result is presented in Fig. 5, which assigns a color for each axial line, according to its value. The RGB color scale follows a continuous function in which blue is the minimum value and red the maximum value.

The nodes which present higher connectivity (node degree) values (see Fig. 5a) concentrate mostly at the center and eastern part of Lisbon network of open public spaces and belong mainly to three communities: Avenidas Novas, the largest community at the geographical center of the map; Parque das Nações, located at the East of the city; and Baixa-Santos, which covers almost the whole historical center of the map and is located at the South, facing Tagus River. The second most connected line links the big void of axial lines (the airport) to the river, passing through the most connected axial line, Alameda dos Oceanos, the main street of Parque das Nações. Both Avenidas Novas and Parque das Nações result from the implementation of urban plans. Baixa-Santos is mainly an organic urban fabric, but also contains different plans from XVII century until the present days.

Based on closeness centrality, global integration measures how close each node is from all the others of the network, while comparing the network to a diamond shaped root one. Therefore, this measure reveals (see Fig. 5b) that the topological center of the network extends from the historical center (Baixa-Santos community), to the geographical center (Av. Novas community) and to the Eastern center of Lisbon (Parque das Nações community). The most integrated axial line corresponds to a segment of Avenida Marechal Craveiro Lopes, located next to the airport, which confirms the efforts made to maximize the road accessibility to this infrastructure.

The global choice values detect the axial lines which present the through-movement potential, as it is a measure based on betweenness centrality. The resulting map (Fig. 5c) highlights the main traffic routes in Lisbon’s urban fabric. It is visible that there are few high choice axial lines in the network, which results in rush hour traffic congestions. The axial line with higher choice value is Av. Marechal Gomes da Costa, which was already referred as the second most connected line. It is also a street which links the airport to the river, as Av. Marechal Craveiro Lopes.

Control value is a local measure, as connectivity, as it is related to the connectivity of the immediate neighbors of each axial line. Therefore, the axial lines with higher control value reveal their importance at the local level, the neighborhood. Although the axial line with higher control value is again Av. Marechal Gomes da Costa, this exposes not its importance to the whole network, as global choice, but its local importance to the immediate neighbors and surroundings. As we can see in Fig. 5d, other axial lines stand out from their neighbors (even not as much as Av. Marechal Gomes da Costa) identifying the most important axial lines in the proximity.

**Fig. 6:**
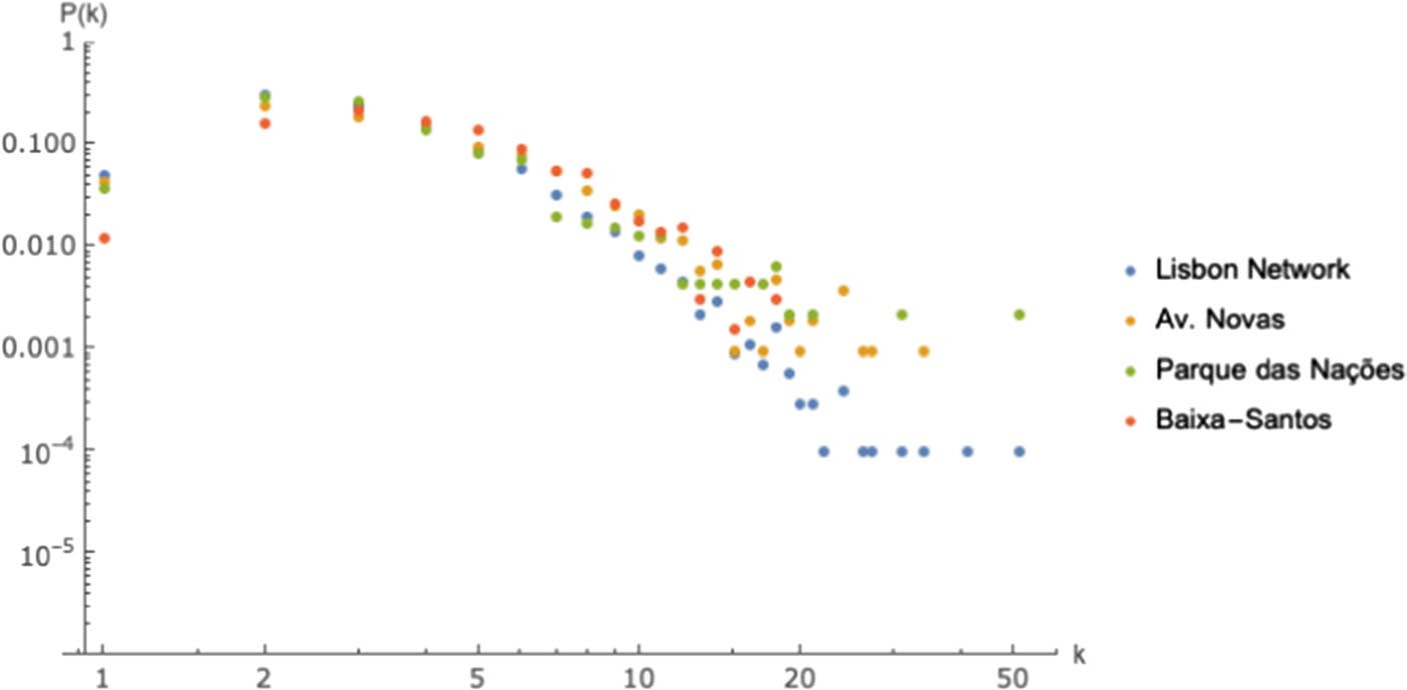
Degree distribution. Comparing the degree distribution for the global network and selected communities

Next, we compared degree and integration topological properties of the whole network with those of three distinct communities, Avenidas Novas, Baixa-Santos and Parque das Nações. The selection of communities was related to their size and structure. Avenidas Novas is the largest community, located at the geographical center of the axial map. It is formed of several grid-like neighborhoods, with different scales and orientations. Baixa-Santos is also one of the largest communities, formed by the most part of the city historical center fabric. It combines irregular (historical center) and regular urban fabrics, along the Tagus river. A comparative analysis of the degree distribution, *P*(*k*), for the whole network and for three selected communities is represented in Fig. 6. Nodes with $k=1$ represent dead-end street segments or limits of the axial map (for the whole network), and are thus less frequent than in typical networks. The high probability of nodes with $k=2$ is a result of the axial map representation, due to the fact that many linear spaces (streets) of the network cannot be represented by a single axial line as they are not completely straight. Therefore, the more irregular the urban fabric, higher the probability of $k=2$. The degree distribution of the nodes for which $k>2$ follows a power law function for both the global network and the three communities. Values drop more rapidly for the whole Lisbon network and are similar for both communities, which is an indicator that the proportion of nodes with higher degree is lower at this level than for the selected communities. Hubs, which were already detected by the assortativity value, can now be observed in the whole network and in Av. Novas community, suggesting that similarly to the global network, this community may contain significant sub-centralities.

**Fig. 7:**
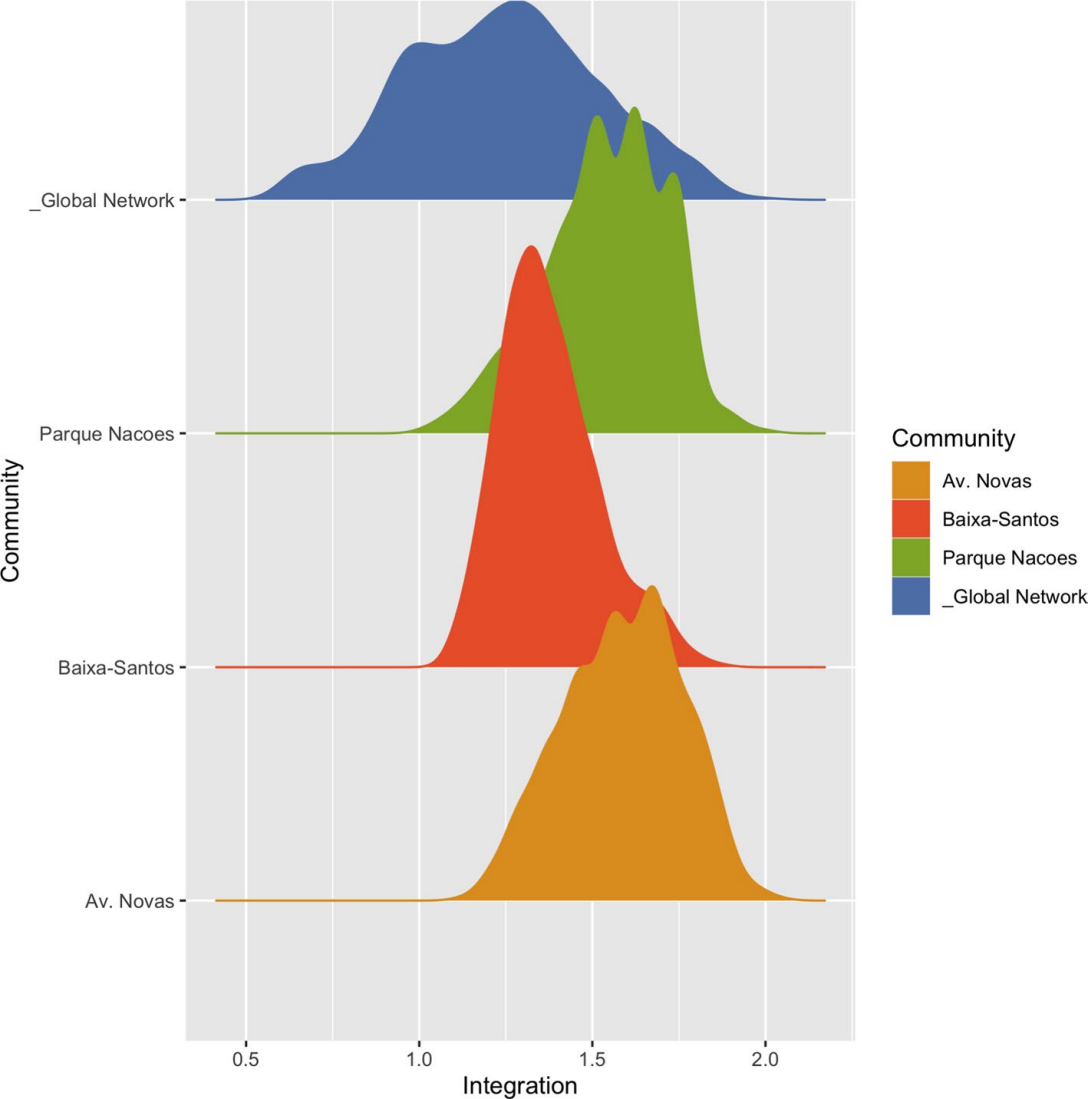
Integration probability density. Comparing the integration distribution for the global network and selected communities

The distribution of integration values for nodes of the global network was also compared with the nodes in the same communities, and is represented in Fig. 7.

The integration distribution suggests that the global network and communities do not exhibit similar characteristics. At the network level, integration values reveal a subtle probability curve, which is higher at the middle and middle-left of the *x* axis. Avenidas Novas presents several stepped peaks, spread along the middle-left part of the *x* axis, which is related to the regularity of the urban fabric and a result of the intentional street hierarchy through urban planning and design of the XIX and XX centuries. Baixa-Santos, on the contrary, reveals a sharp peak at the left, located at $Integration_{(i)} = 1.38$, which is higher than the average integration of the whole network ($Integration_{(i)} = 1.24$), followed by a longer right tail. This distribution suggests that, although there are some nodes with high integration values, most of the nodes have an average value considering the whole network. Higher integration nodes are rare and structural to the life of the community, playing the role of places of encounter.

**Fig. 8:**
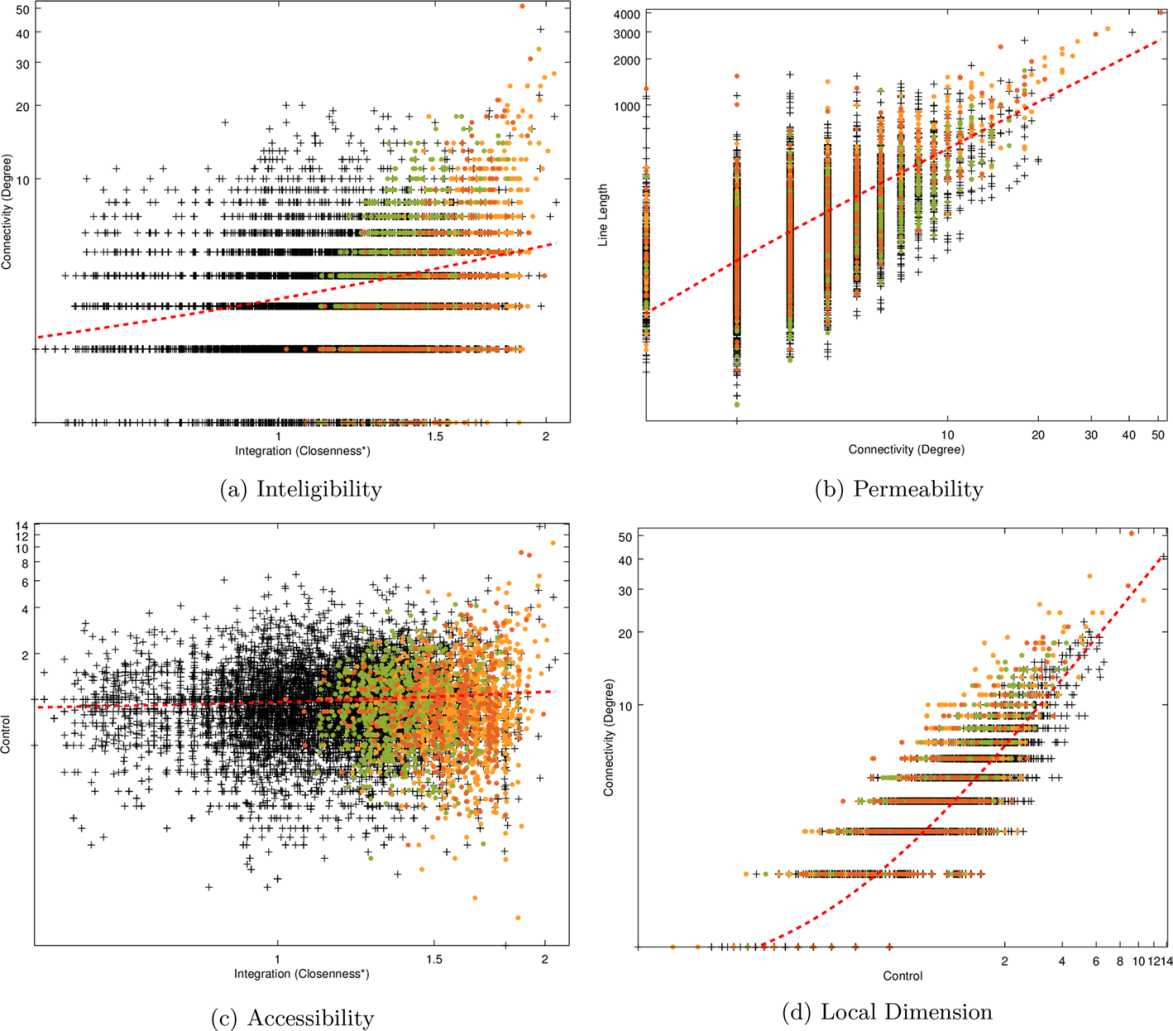
Space Syntax second order measures for the whole network and three selected communities: Av. Novas, Parque das Nações and Baixa-Santos

The results for the network second order measures can be visualized in Fig. 8.

Intelligibility (see Fig. 8a) presents an $R = 0.2331$, which is low, as expected for the global scale. However, when we see the colored points in the scattergram for three communities, it is clear that the regression line will present a steeper angle, especially for Avenidas Novas community. One can infer that an individual who is located on an axial line belonging to one of the observed communities can easily ’read’ the whole community from its vision angle. However, the whole city is a much more diverse system and cannot be totally apprehended from a single location.

Permeability (see Fig. 8b) seems to be a more consistent measure at the global and community scale, as the observed communities are distributed along the regression line, except Avenidas Novas, which presents more axial lines at the higher values. The *R* value is high for the global network ($R = 0.6880$), which means that even when axial lines are long, there is a variety of alternative paths to choose. Urban fabrics with natural obstacles or large blocks tend to present lower values.

Accessibility scattergram (see Fig. 8c) presents no significant correlation between global integration and control value for the whole network ($R = 0.0654$). The three observed communities are located at the right of the graphic, because of the higher average values of global integration (see Fig. 7), but similar to the whole network, do not present correlation with control. The reason for this accessibility values is that the major part of axial lines with high control value present lower values of integration, that is, are more peripheral to the network, although being locally important to their immediate neighbors.

Local dimension (see Fig. 8c) presents a high *R* value ($R = 0.8087$) for the whole network. However, similar to intelligibility, the three communities present steeper regression line angles when compared to the global network. This can be interpreted as the sense of safety is generally present at the global scale, but accentuates particularly in these communities.

**Fig. 9:**
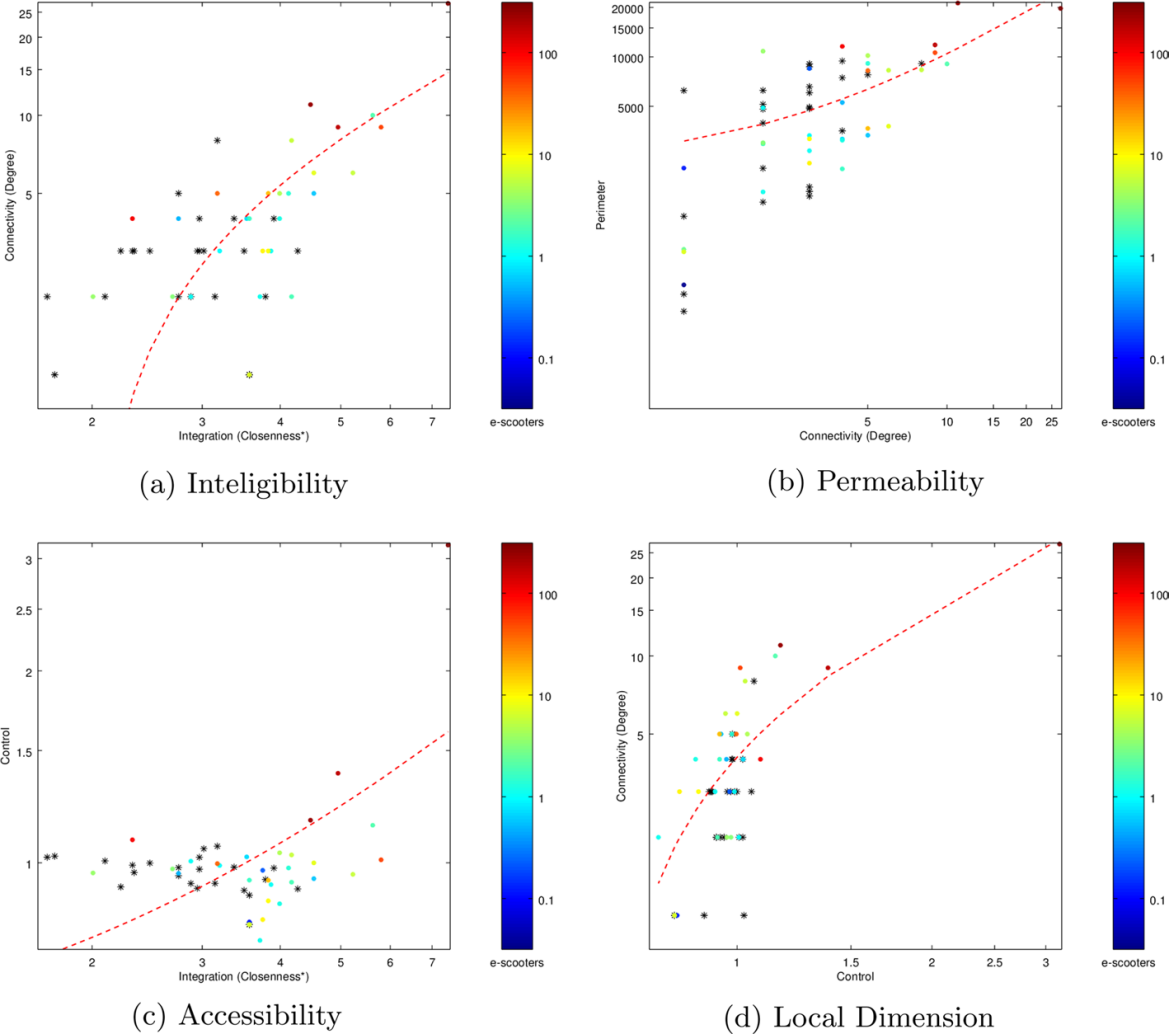
Space Syntax second order measures using communities as nodes. Colours indicate the average number of e-scooters found in each community (* for 0 e-scooters)

To assess the relations between communities, we used the communities’ graph (see Fig. 3), taking each community as a node. Every inter-community connection remained and the average number of e-scooters within each community was calculated. Figure 9 presents the second order measures for this level of analysis.

In general, all measures correlate better at this level than for nodes in the whole network, especially for intelligibility ($R = 0.7087$; see Fig. 9a) and accessibility (see Fig. 9c). In the latter case, the improvement is from almost none correlation, to an acceptable value of $R = 0.5027$, which means that the most accessible communities (global integration) also have control over the immediate neighbor communities. Moreover, it is noticeable that the communities with greater average number of e-scooters usually also present higher values of each measure. However, there are some cases with lower values and high number of e-scooters, which means that although average agglomeration of e-scooters is highly related with the topological properties of the network, other factors that justify their presence are still missing to find.

**Fig. 10:**
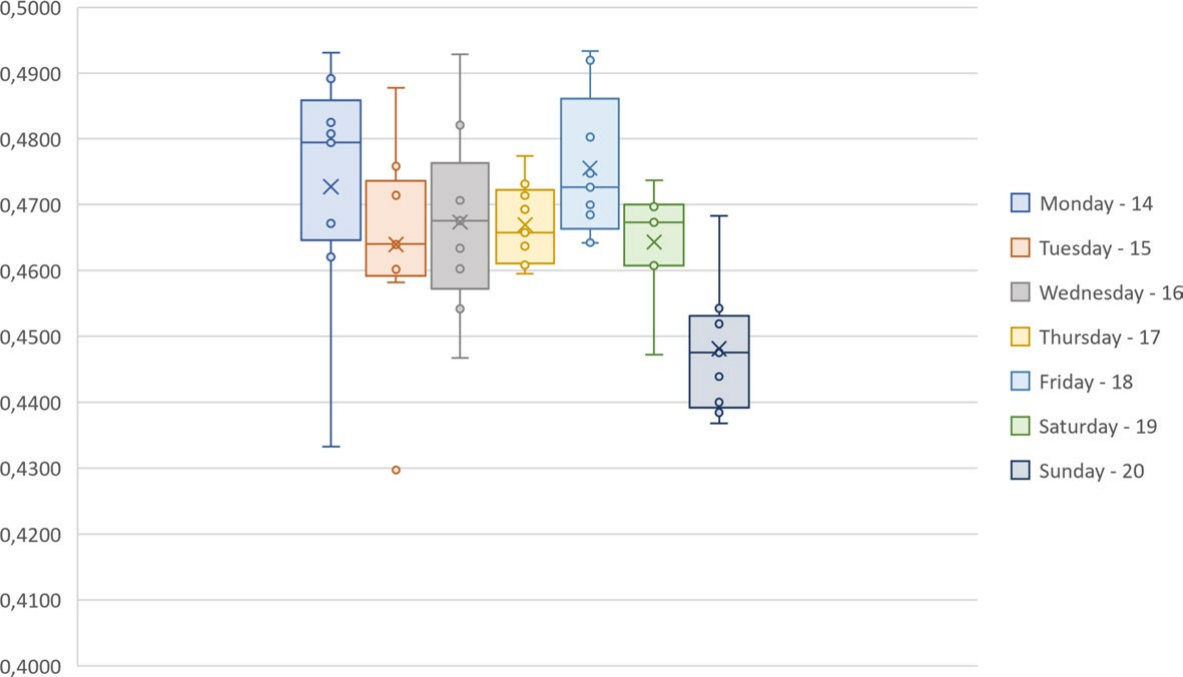
Variation of Information (VI) between network communities and e-scooters’ clusters Boxplot of VI for each day of the sample week

Regarding the VI between network communities and e-scooters’ clusters through time (see Fig. 10), we can observe that there is no moment during the sampled time in which the e-scooters assemble is fully coincident with the community structure of the network. Actually, the days when VI is lower are weekend days, when mobility restrictions due to COVID-19 are stronger (after 13 h). This observation may indicate that e-scooters are relocated in clusters more similar to network communities, but their actual use resumes the difference. The higher peaks of average VI where observed during mornings and after lunchtime (13-15h), which are the expected hours of more public space fruition, especially during the pandemic.

**Fig. 11:**
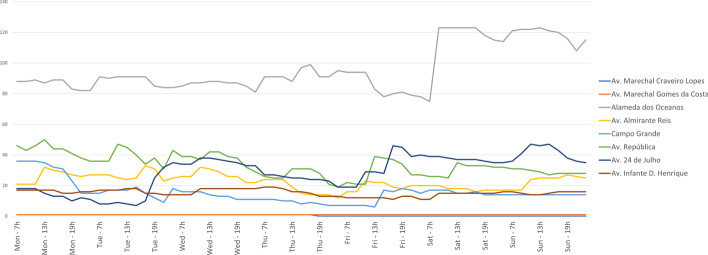
Variation of the number of parked scooters in some axial lines through time The selected axial lines present higher values in first order measures on the network and/or community levels

To examine temporal variations at the local level, we selected some of the axial lines which presented higher values of space syntax measures and observed the evolution in the number of parked scooters (see Fig. 11).

Although Av. Marechal Craveiro Lopes is the most integrated line of the network, and Av. Marechal Gomes da Costa presents the highest value of global choice and control value and the second in connectivity, they seem to be of no interest for users to pick/drop e-scooters. In fact, global integration and choice are two measures of accessibility to the whole system, which can be interpreted as more suitable for longer travels and therefore, for other means of transportation, as cars. Also, other factors may interfere on the decision to use e-scooters, as sidewalk width, asphalt/sidewalk area ratio, existence of attractors (as local commerce), cycle paths or land use.

Alameda dos Oceanos is the most connected line of the network, while also being a positive outlier in local dimension and it seems to be perceived as attractive both to users and management entities of e-scooters, as it stands out for the supply number of these vehicles, while presenting consistent variation through time, meaning that there is a permanent number of e-scooters leaving and entering that space.

The peaks of supply, starting Friday afternoon and during the weekend, are consistent with observed lower values of VI, as the selected axial lines play significant roles in the network topological relations.

At the community level, Av. 24 de Julho stands out for its stronger variations on Tuesday afternoon and Sunday morning. This axial line represents a long, scenic street along Tagus river, with several touristic attractions, green areas and cycle paths, while being structural to the local area accessibility. Thus, it suggests that other factors such as routine habits of sports and leisure or touristic and cultural events have a strong influence on the use of public space and movement patterns. This is noticed particularly when compared to the most significant axial lines of Avenidas Novas community, a commercial and business center of Lisbon, Av. Almirante Reis, Campo Grande and Av. República, whose variations on e-scooters number is much more constant.

## Conclusions, limitations and future work

We have investigated the relations between topological properties of the network of open public spaces of Lisbon and the use of public space by micromobility vehicles. We have analyzed first and second order measures developed in space syntax theory, which revealed the structural factors that may determine movement patterns in the studied network.

The research started with the study of the global properties of the network, the macrolevel analysis, which confirmed that Lisbon’s open public spaces network is sparse, its degree distribution follows mostly a power law, as many other studied real networks, presenting hubs, or highly connected nodes, which allows some hierarchical structure and local areas of intense living.

Data collected from e-scooters locations during one week confirmed that spatial morphology is an important factor conditioning human behavior although not unique; other factors may coexist, such as spatiotemporal cycles, events or qualitative factors.

At the mesolevel, we detected communities based on the topological properties of the network, and compared this partition with the partition of e-scooters locations, which revealed that they were not fully coincident, suggesting that other factors beyond spatial structure may also determine the use of public space.

Although authors focused on the case study of Lisbon, the same methodology can be applied to other cities and compare different cultural behaviors in other urban environments.

One of the main difficulties in generating axial maps is the definition of the lines, which can be time consuming and the definition criteria argued as ambiguous. Several methodologies and algorithms have been developed, but large networks are still hard to compute. Therefore, the axial map used for the study will be updated for the most recent urban developments to continue the research.

Other limitations concern the micromobility data. The anonymization of e-scooters ID’s was an obstacle to analyze the movement patterns of these vehicles, as for each moment of data collection, new ID’s were generated, preventing route tracking. Also, the collection timeframe coincided with social and economic activities restrictions due to COVID-19 pandemic. Therefore, the results of the study reflect this circumstances. Further studies will be conducted, when public life comes back to the urban space.

Improvements on the analysis of factors conditioning movement patterns in the public space, such as including land uses, topography and other spatial data sources will be developed in further research. In addition, other micromobility data sources, pedestrian movement (from mobile data), and public transport will be included via multilayer networks analysis, as this is a critical issue to produce a transversal analysis of mobility patterns.

Possible extensions of space syntax to an hypernetwork representation and Q-analysis could also improve structural analysis of movement patterns.

## Data Availability

The axial map data that support the findings of this study are available from Prof. Francisco Serdoura (CIAUD, FAUL) but restrictions apply to the availability of these data, which were used under license for the current study, and so are not publicly available. Data are however available from the authors upon reasonable request and with permission of Prof. Francisco Serdoura (CIAUD, FAUL). E-scooters’ positional data is available via Bird’s API at https://mds.bird.co/gbfs/system_information.json.

## Abbreviations

AC: assortativity coefficient
API: application programming interface
CAD: computer aided-design
CV: control value
GIS: geographic information systems
GPS: global positioning system
RGB: red, green, blue
RRA: real relative asymmetry
VI: variation of information

## References

[CR1] Arthur D, Vassilvitskii S (2007) k-means++: the advantages of careful seeding. In: SODA ’07: proceedings of the eighteenth annual ACM-SIAM symposium on discrete algorithms. Society for Industrial and Applied Mathematics, Philadelphia, PA, USA, pp 1027–1035

[CR2] Barabási A-L, Pósfai M (2016). Network science.

[CR3] Barthelemy M (2019). The statistical physics of cities. Nat Rev Phys.

[CR4] Batty M (2013). The new science of cities.

[CR5] Batty M (2018). Digital twins. Environ Plan B Urban Anal City Sci.

[CR6] Bettencourt L, West G (2010). A unified theory of urban living. Nature.

[CR7] Bird e-scooter API. https://mds.bird.co/gbfs/system_information.json

[CR8] Boeing G (2019). Urban spatial order: street network orientation, configuration, and entropy. Appl Netw Sci.

[CR9] Clauset A, Newman MEJ, Moore C (2004). Finding community structure in very large networks. Phys Rev E.

[CR10] Dhanani A, Tarkhanyan L, Vaughan L (2010). Estimating pedestrian demand for active transport evaluation and planning. Transp Res Part A.

[CR11] Fortunato S (2010). Community detection in graphs. Phys Rep.

[CR12] Gallotti R, Bertagnolli G, Domenico MD (1993). Unraveling the hidden organisation of urban systems and their mobility flows. EPJ Data Sci.

[CR13] Guo W, Doñate GM, Law S, Johnson S, Liakata M, Wilson A (2017) Urban analytics: multiplexed and dynamic community networks. CoRR arXiv:abs/1706.05535

[CR14] Hillier B (1996). Space is the machine: a configurational theory of architecture.

[CR15] Hillier B (2009) Spatial sustainability in cities: organic patterns and sustainable forms. In: Koch D, Marcus L, Steen J (eds) Proceedings of the 7th international space syntax symposium; Stockholm, p KTH

[CR16] Hillier B, Penn A, Hanson J, Xu J (1993). Natural movement: or, configuration and attraction in urban pedestrian movement. Environ Plan B Plan Des.

[CR17] Jacobs J (1993). The death and life of Great American cities.

[CR18] Klarqvist B (1993). A space syntax glossary. Nordisk Arkitekturforkning.

[CR19] Koohsari M, Oka K, Owen N, Sugiyama T (2019). Natural movement: a space syntax theory linking urban form and function with walking for transport. Health Place.

[CR20] Kruger MT (1989) On node and axial grid maps: distance measures and related topics. In: Unit for Architectural Studies UCL (ed) European conference on the representation and management of urban change, pp 1–34

[CR21] Kruger M, Vieira AP (2012). Scaling relative asymmetry in space syntax analysis. J Space Syntax.

[CR22] LeFebvre H (2004). Elements of rhythmanalysis: an introduction to the understanding of rhythms.

[CR23] Liu X, Jiang B (2012). Defining and generating axial lines from street center lines for better understanding of urban morphologies. Int J Geogr Inf Sci.

[CR24] Meila M (2007). Comparing clusterings-an information based distance. J Multivar Anal.

[CR25] Newman MEJ (2002). Assortative mixing in networks. Phys Rev Lett.

[CR26] Newman MEJ (2004). Fast algorithm for detecting community structure in networks. Phys Rev E.

[CR27] Newman MEJ, Girvan M (2004). Finding and evaluating community structure in networks. Phys Rev E.

[CR28] Orman GK, Labatut V (2009) A comparison of community detection algorithms on artificial networks. In: Hal (ed) International conference on discovery science, pp 242–256

[CR29] Penn A (2001) Space syntax and spatial cognition. or, why the axial line? In: Peponis J, Wineman J, Bafna S (eds) Proceedings of the 3rd international space syntax symposium in Atlanta, Geogia, USA, pp 11–11117

[CR30] Penn A, Hillier B, Banister D, Xu J (1998). Configurational modelling of urban movement networks. Environ Plan B Plan Des.

[CR31] Serdoura F (2006) Espaço público, vida pública - o caso do parque das nações. Ph.D. thesis, Instituto Superior Técnico, Universidade Técnica de Lisboa

[CR32] Syakur MA, Khotimah BK, Rochman EMS, Satoto BD (2018). Integration k-means clustering method and elbow method for identification of the best customer profile cluster. IOP Conf Ser Mater Sci Eng.

[CR33] Turner A (2007). From axial to road-centre lines: a new representation for space syntax and a new model of route choice for transport network analysis. Environ Plan B Plan Des.

[CR34] Volchenkov D, Blanchard P (2007) City Space Syntax as a complex network. Phys Soc, ArXiv

[CR35] Yamu C, van Nes A, Garau C (2021). Bill Hillier’s legacy: space syntax-a synopsis of basic concepts, measures, and empirical application. Sustainability.

[CR36] Yildirimoglu M, Kim J (2017). Budapest, Hungary identification of communities in urban mobility networks using multi-layer graphs of network traffic. Transp Res.

